# Tanshinone II Aattenuates renal damage in STZ-induced diabetic rats via inhibiting oxidative stress and inflammation

**DOI:** 10.18632/oncotarget.16651

**Published:** 2017-03-29

**Authors:** Xia Chen, Rui Wu, Yiwei Kong, Yuting Yang, Yu Gao, Dandan Sun, Qizhen Liu, Dongjun Dai, Zeyuan Lu, Niansong Wang, Sheng Ge, Feng Wang

**Affiliations:** ^1^ Department of Nephrology, Shanghai Jiao Tong University Affiliated Sixth People's Hospital, Shanghai 200233, China; ^2^ Department of Nephrology, Shanghai Eighth People's Hospital, Shanghai 200233, China; ^3^ Department of Clinical Nutrition, Shanghai Jiao Tong University Affiliated Sixth People's Hospital, Shanghai 200233, China

**Keywords:** tanshinone, kidney, diabetes

## Abstract

Oxidative stress and inflammation have been demonstrated to be involved in the onset and promotion of diabetic nephropathy (DN).Tanshinone IIA (Tan) possesses both antioxidant and anti-inflammatory properties. Here, the aim of the present study was to explore whether Tan could attenuate renal damage in the rats with streptozotocin (STZ)-induced diabetes and its potential mechanisms. Tan was gavaged to STZ-induced diabetic rats at the dose of 10mg/kg once a day for 12 weeks. Tan treatment significantly attenuated albuminuria and renal histopathology in diabetic rats. Besides, Tan treatment also effectively inhibited oxidative stress and inflammatory reaction in the kidneys of diabetic rats. Our study provided evidence that the protective effect of Tan on diabetes-induced renal injury is associated with inhibition of oxidative stress and inflammation. Tan may be a potential candidate for the treatment of DN.

## INTRODUCTION

Diabetic nephropathy (DN) prevalence is increasing with the expanding size of the diabetes population around the world. It was reported that one-third of patients with DM develop DN [1]. Currently, DN has become leading cause of chronic kidney disease (CKD) in the developed world. The existing treatment for DN are mainly focusing on glycaemic, lipid and blood pressure control, and life-style changes [2]. Despite of those therapeutic approach, nearly 50% patients with DN unavoidably processed to the end stage renal disease (ESRD) in the United States [3], which not only brought along the physical pains for the patients, but also lead to heavy social and economic burden. Therefore, it is urgent to explore the mechanisms and the novel treatments for DN [4, 5].

Tanshinone IIA (Tan) is one of the major diterpenoids derived from Salvia miltiorrhiza (S. miltiorrhiza) [6] referred to one of the two lipophilic components of ‘Danshen’ [7], which also contains hydrophilic components. Tan exhibited protective effects on several pharmacological targets, including anti-oxidative stress [8], anti-inflammation [9] and anticancer [10]. A large number of reports demonstrated Tan has been used for the treatment of multiple diseases, including cardiovascular disease [11], cerebral ischemia [12] and bone diseases such as osteoporosis [13]. In kidney disease, Tan not only protects against acute kidney injury [14, 15], but also has protective effects on chronic kidney disease, including uric acid nephropathy [16], doxorubicin induced nephropathy [17], 5/6 nephrectomy induced chronic kidney disease [18] and hypothermic kidney preservation [19]. Although Kim et al. [20] demonstrated that Tan exerted protective effect on the early stage of diabetic nephropathy in animal model, the mechanisms remain largely unknown.

Therefore, in the current study, we have designed animal experiments to determine the therapeutic action of Tan and investigate its potential mechanisms in renal injury induced by streptozotocin (STZ) type 1 diabetes using rats. This study aims at exploiting to develop the underlying therapy for DN.

## RESULTS

### Tan alleviated albuminuria in diabetic rats

**Figure 1:**
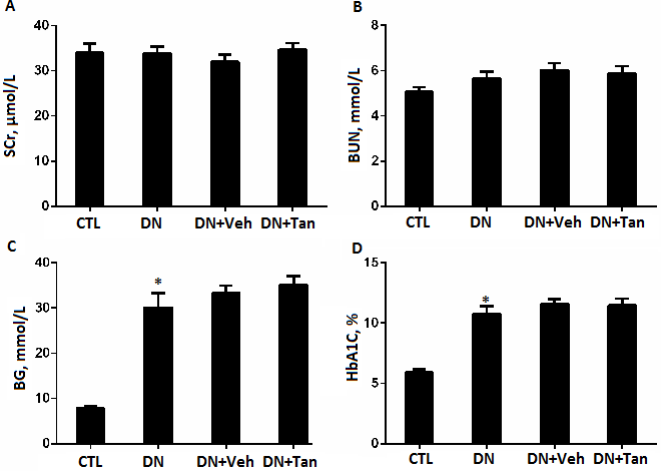
The effect of Tanshinone IIA on renal function and blood glucose in STZ-Induced diabetic rats (**A**) serum creatinine (SCr); (**B**) blood urea nitrogen (BUN); (**C**) blood glucose (BG) and (**D**) hemoglobin A1C (HbA_1_C).CTL, normal non-diabetic rats; DN,STZ-induced diabetic rats; DN+Veh, DN rats treated with vehicle, DN+ Tan, DN rats treated with Tanshinone IIA. Results are expressed as means ± SD (*n* = 6/each group,*P < 0.01, versus CTL group).

**Figure 2:**
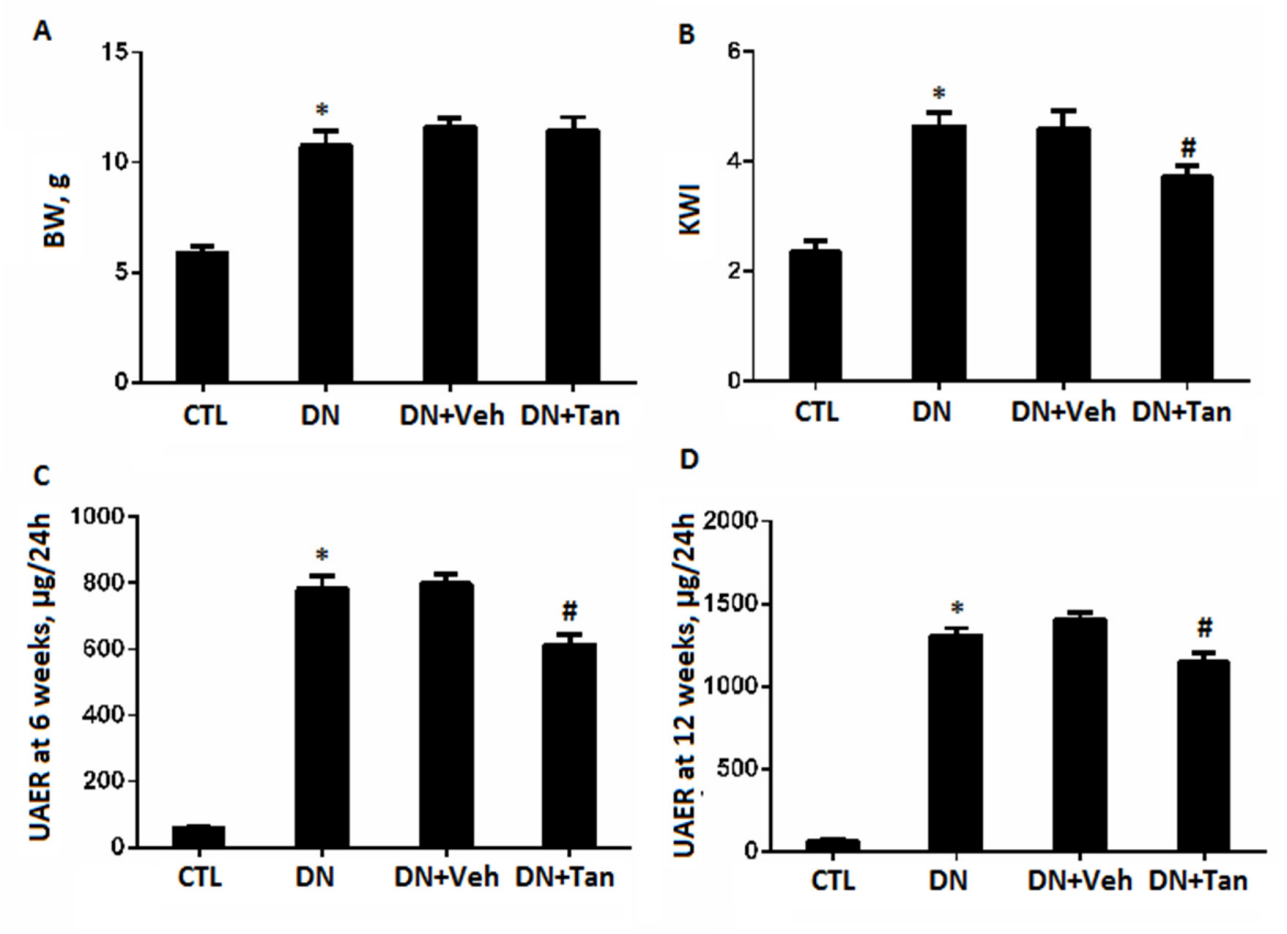
The effect of Tanshinone IIA on biochemical parameters in STZ-Induced diabetic rats (**A**) body weight; (**B**) ratio of kidney weight to body weight; urine albumin excretion at the end of 6 (**C**) and 12 weeks (**D**). CTL, normal non-diabetic rats; DN,STZ-induced diabetic rats; DN+Veh, DN rats treated with vehicle, DN+ Tan, DN rats treated with Tanshinone IIA. Results are expressed as means ± SD (*n* = 6/each group,*P < 0.05, versus CTL group.^#^*P* < 0.05,vs DN+Veh group).

There were no significant changes about body weight, blood glucose levels, HbA1c level and renal function including serum creatinine (SCr) and blood urea nitrogen (BUN) among the diabetic groups Figure 1. Albuminuria is considered as a hallmark for the progression of renal disease [21]. Tan significantly decreased urinary albumin excretion (UAE) in diabetic rat, as show in Figure 2, DN rats treated with Tan had greatly reduced proteinuria after treatment for 6 wk and 12 wk (*P* < 0.05). Amazingly, the ratio of the kidney weight to body weight (KWI) in DN rats received Tan treatment was lower compared with DN group and vehicle group (*P* < 0.05). These data suggested that Tan could potentially protect the kidneys in diabetic rats, which was independent of blood glucose decrease.

### Tan attenuated renal histopathology injury

**Figure 3:**
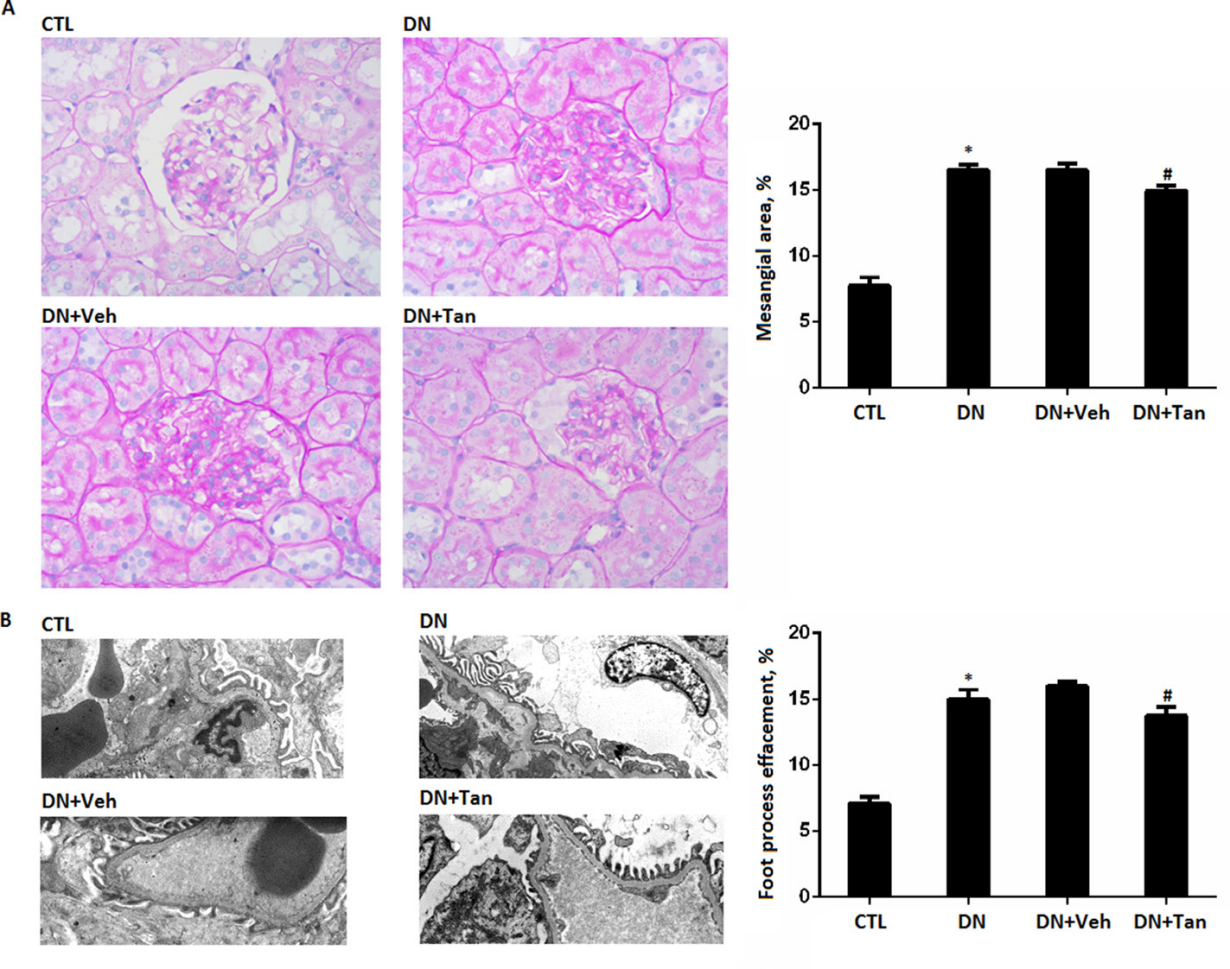
The effect of Tanshinone IIA on morphology change in diabetic rats (**A**) PAS staining of renal sections (400X); Quantification shows the percentage of mesangial area in the glomeruli of rats from each group. (**B**) the glomerular ultrastructure (Original magnification 3500X); Quantification shows the percentage of foot process effacement in the glomeruli of rats from each group. CTL, normal rats; DN, STZ-induced diabetic rats;DN+Veh, DN rats treated with vehicle, DN+ Tan, DN rats treated with Tanshinone IIA. Data are expressed as means ± SD (*n* = 6/each group). *P < 0.05, versus CTL group.^#^*P* < 0.05,versus DN+Veh group.

Morphologically, DN is defined by thickening of the glomerular basement membrane (GBM) and mesangial expansion, contributing to a progressive damage in the glomerular filtration barrier [22]. As shown in Figure 3A, the diabetic rats showed focal mesangial matrix expansion compared to non-diabetic rats. However, Tan treatment significantly decreased the percentage of mesangial expansion area (Figure 3A) (*P* < 0.05). To assess foot process effacement in the diabetic kidney, the tissue sections were observed by electron microscopy.There was a widespread foot process fusion in the kidneys of diabetic rats. After Tan treatment for 12 weeks, the diabetic rats showed decreased foot process fusion when compared with the non-treated rats (*P* < 0.05) (Figure 3B). Therefore, these data suggested Tan attenuated renal histopathology injury in DN rats.

### Tan ameliorated the inflammatory reaction in the kidneys of STZ-induced diabetic rats

**Figure 4:**
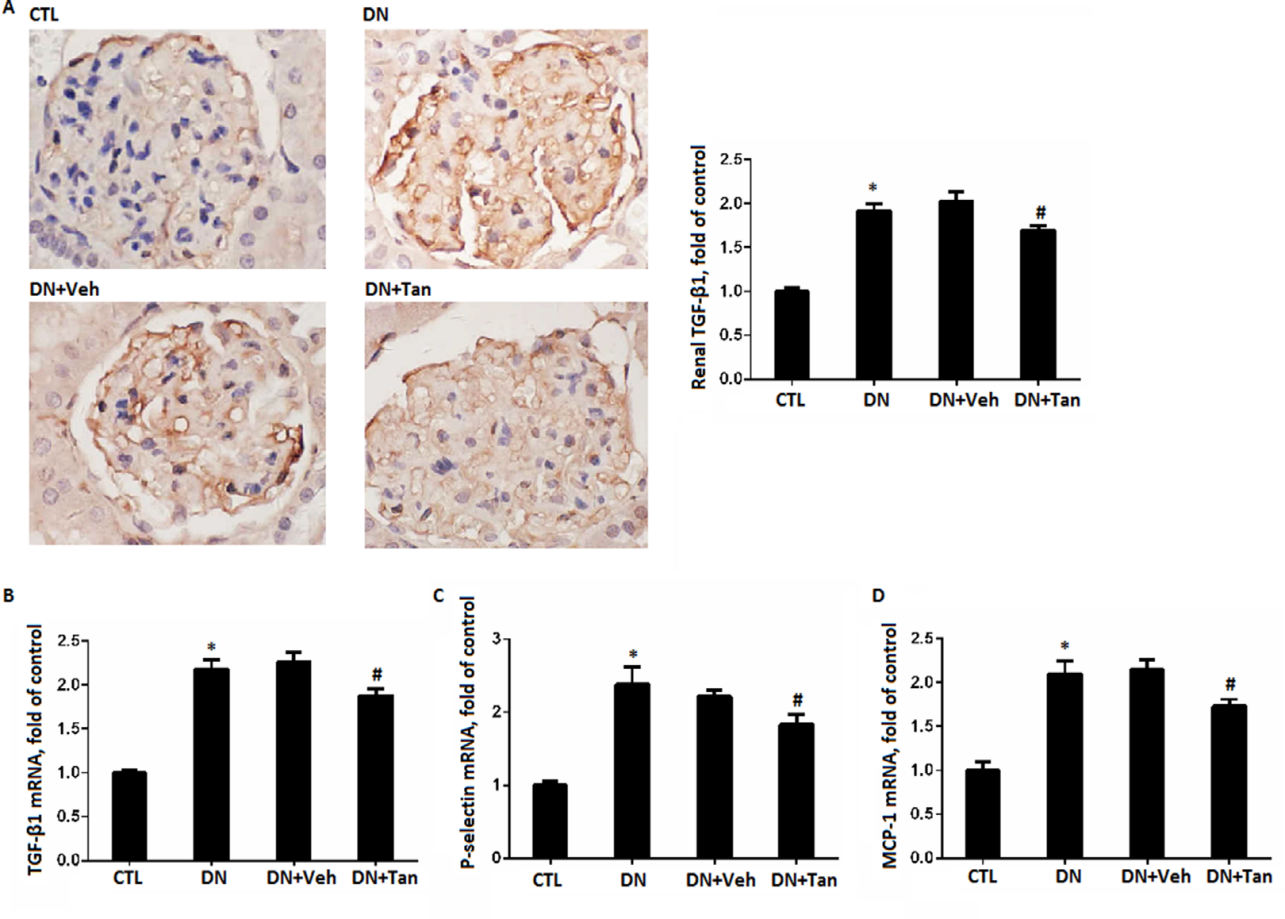
Tanshinone IIA reduced the inflammation (**A**) Immunohistochemistry staining for TGF-β1 in renal sections (Original magnification 400X) and semi-quantitative data TGF-β1 staining in different groups of rats. Gene expression of TGF-β1 (**B**), P-selectin (**C**) and MCP-1 (**D**) by real-time PCR analysis of kidney cortex from rats in different groups. Data are expressed as means ± SD. *P < 0.05 versus CTL group, ^#^*P* < 0.05 versus DN+Veh group, *n* = 6.

Inflammation played an important role in the pathogenesis of diabetes [23, 24]. As shown in Figure 4, immunohistochemistry assay showed increased TGF-β1 expression in the renal cortex from STZ-induced diabetic rats, which decreased by Tan (*P* < 0.05). Coincidentally, we found Tan treatment also reduced the gene level of TGF-β1 in renal cortex of DN rats using real time PCR assay (*P* < 0.05) (Figure 4B).In addition, mRNA expression of P-selectin and MCP-1 were decreased in Tan-treated diabetic rats correspondingly (*P* < 0.05). Taken together, the above results indicated that Tan treatment ameliorated diabetes-induced inflammatory reaction in kidney of DN rats.

### Tan inhibited the renal oxidative stress and serum CRP in DN rats induced by STZ

**Figure 5:**
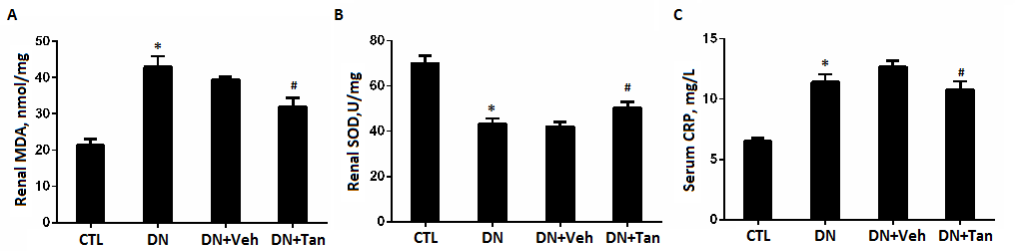
Tanshinone IIA inhibited the renal oxidative stress markers and serum CRP in DN rats Quantitative data of renal MDA (**A**) and SOD (**B**) and serum CRP (**C**) in different groups of rats. Data are expressed as means ± SD. *P < 0.05 versus CTL group, ^#^*P* < 0.05 versus DN+Veh group, *n* = 6.

Oxidative stress is one serious factor in promoting DN. To investigate the potential renoprotective mechanism of Tan in DN, we investigated the effects of Tan on oxidative stress in kidney of DN rats. As shown in Figure 5A–5B, the concentration of MDA was remarkably increased and the activity of SOD was significantly reduced in DN rats, which were reversed by Tan (*P* < 0.05). Compared with non-diabetic rats group, the concentration of serum CRP in DN group increased significantly (Figure 5C), which were also prevented by Tan effectively (*P* < 0.05).

## DISCUSSION

In this study, we re-confirmed that Tan treatment could protect against early stage diabetic nephropathy in the rats with diabetes induced by STZ. Our data revealed that Tan reduced albuminuria and KWI and alleviated renal histopathological injury in diabetic rats. The oxidant/antioxidant balance was observed by the decrease of MDA and the increase of SOD in kidneys of STZ-induced diabetic rats received with Tan. Moreover, reduced inflammatory cytokines and profibrogenic mediators including MCP-1, TGF-β1, P-selectin and CRP also were observed either in the renal cortex or serum of Tan-treated diabetic rats. However, Tan treatment has no effect on blood glucose and HbA1c in diabetic rats. These data indicate that Tan may exert its reno-protective effects through anti-oxidation and anti-inflammation, which was not relevant to blood glucose decrease.

Clinically, DN was characterized by microalbuminuria (> 300 mg/24 h). As DN evolves, macroalbuminuria and renal disfunction take place [25]. Our results suggested the renal function including SCr and BUN made no significant difference in rats with or without DN, which indicated diabetic rats exhibited mild and early diabetic kidney injury at 12 wk post STZ injection. Further Tan treatment also exerted no effect on renal function, which indicated Tan had no toxicity to kidney in rats at the early stage of DN. However, Tan treatment reduced albuminuria and delayed the development of DN, which is consistent with previous report [20]. Therefore, Tan attenuated early renal damage in diabetic rats induced by STZ.

Despite the fact that the exact mechanisms of DN are not clear yet, poor glucose control and the activators of oxidative stress and inflammation are proposed to promote the progress of DN from early stage to advanced stages [26]. In the present study, we found that the renoprotective effect of Tan on DN is not mediated by decreasing blood glucose. Histopathologically, Tan significantly reduced the accumulation of extracellular matrix (ECM) and fibrocytes in the diabetic kidneys, which might be important mechanisms underlying the beneficial effects of Tan on DN. Activation of cytokines, profibrotic and inflammatory factors might be involved in the ECM accumulation that takes place in DN.

TGF-β1 is a well-known cytokine mediated cell hypertrophy, increased synthesis of collagen contributing to ECM accumulation, leading ultimately to glomerulosclerosis in diabetic nephropathy [27, 28]. Previous reports have demonstrated that Tan can suppress TGF-β1 expression *in vitro* [29] and *in vivo* [15]. Similarly, Our results also shown that Tan can inhibit TGF-β1 expression in renal cortex of diabetic rats induced by STZ. In addition, MCP-1 has been also demonstrated to be directly involved in extracellular matrix (ECM) synthesis [30] and exerted its proinflammatory effect [31] in the pathogenesis of diabetic nephropathy. Our data suggested that glomerular MCP-1 expression is increased in experimental diabetic rats, which is in line with previous studies [32, 33]. Treating with Tan could downregulated MCP-1 expression and reduced the accumulation of ECM. Taken together, Tan was likely effective in reducing ECM accumulation in STZ-induced diabetic rats.

Many studies have demonstrated that Tan has anti-inflammatory effect and is applied in inflammatory diseases such as arthritis, hepatitis and endangitis [9]. P-selectin has been demonstrated to play an important role in the context of inflammatory diseases [34, 35]. Our data also reconfirmed P-seletin was increased in the DN rats induced by STZ, which is consistent with our previous study. Wang et al. reported Tan attenuated inflammation in 5/6 nephrectomized rats [36]. In agreement, our data also demonstrated that Tan administration alleviated inflammatory factor such as CRP,P-selectin and MCP-1 in DN rats. The above data reconfirmed Tan protecting against diabetes induced renal injury is partly associated with its anti-inflammation.

Besides, DN is an oxidative-stress related microvascular disease [37]. Hyperglycemia is known to induces oxidative stress that is due to disturbance of oxidant/antioxidant balance, playing a critical role in kidney damage [38]. The antioxidant treatment may be important to optimize renoprotection in diabetes or other kidney disease [39–42]. In our study, Tan administration was shown to decrease MDA level and increase SOD level due to its potential antioxidant property, which suggested that Tan depressed oxidative stress in mediating glucose-induced renal injury.

In summary, Tan treatment in this study decreased 24-h urine protein excretion and KWI and attenuated renal histopathology in a STZ-induced diabetic rat model. Further, Tan significantly inhibited the expressions of oxidative stress and inflammation related markers in this model. Therefore, this work suggested that Tan delayed the promotion of DN via inhibiting oxidative stress and inflammatory reaction, which offers novel therapeutic targets that can be exploited to develop new renoprotective treatments for DN in the future [43–44].

## MATERIALS AND METHODS

### Animals

Male Sprague-Dawley rats (weighing 250 ± 20 g) were purchased from Shanghai Science Academy Animal Center (Shanghai, China) and housed in the SPF room where the temperature was about 22 ± 1°C.All the rats were allowed access to food and water ad libitum with a constant light cycle (12 h light/dark). This animal study was approved by the Laboratory Animals Ethical Committee of Sixth People's Hospital Affiliated to Shanghai Jiao Tong University, China.

### Experimental design

After one week adaptation, rats were intraperitoneal injected with streptozotocin (STZ,Sigma-Aldrich, St. Louis, MO, USA) at 65 mg/kg to induce diabetes (DN, *n* = 6). Control rats were administrated equal amount of vehicle (0.1 M citrate buffer, pH 4.6) by intraperitoneal injection (Control, *n* = 6).72 hours later, the blood glucose level from tail veil over 16.7mM was considered as diabetes. Then, the diabetic rats were randomly divided into 3 groups: DN group without treatment (DN, *n* = 6); DN+Tan group gavaged with Tan (10 mg/kg,Jiangsu Carefree Group Co.,Nanjing, China) in corn oil once a day (DN+Tan, *n* = 6); DN+Veh group treated by gavage with same amount of corn oil (DN+Veh, *n* = 6). The dosage of treatment was based on the body weight of the rats. All the animals were sacrificed 12wk after the treatment of Tan.

### Urinary albumin excretion

All the rats were housed in individual metabolic cages for 24 h urine collection at the end of 6 and 12 weeks of treatment for the evaluation of urine albumin excretion rate (UAER).

### Blood samples collection and determination

Rats were anesthetized by intraperitoneal injection of pentobarbital sodium and the blood samples were immediately taken through abdominal aorta for measuring biochemical parameters, including the blood urea nitrogen (BUN) and serum creatinine (SCr) by ELISA kits (Nanjingjiancheng, Nanjing, Jiangsu, China) according to the manufacturer’ instructions and HbA1c by HPLC method. The ratio of kidney weight to bodyweight was considered as kidney weight index (KWI).

### Morphologic analysis

The histological analysis was conducted similar to that described previously [45–47]. Briefly, 10% formaldehyde solution-fixed paraffin-embedded renal tissues were stained with periodic acids chiff (PAS) and assessed by light microscopy for levels of mesangial area and 2.5% glutaraldehyde-fixed renal tissues were assessed by electron microscope for ultrastructural changes. All the sections were examined by two pathologists in a blind manner.

### Immunohistochemistry

To detect the renal cortex expression of TGF-β1, immunohistochemical assay was performed similar to that described previously [48–50]. Anti-TGF-β1 rabbit monoclonal antibody and horseradish peroxidase-conjugated secondary antibody were purchased from Shanghai Immune Biotech, China. The Immunostaining of TGF-β1 was quantified by ImageProPlus Systems.

### Oxidative stress and inflammation biomarkers

Malondialdehyde (MDA) level and superoxide dismutase (SOD) level in renal tissues were measured by the commercial kits (A001–1 and A003–1, Nanjing Jiancheng Bioengineering Institute, Jiangsu, China) using spectra microplate reader (model A-5082, Tecan, Australia), similar to that described previously [51–53]. C-reactive protein (CRP) was also measured in serum of rats using a commercial assay kits (Shanghai Immune Biotech, China), according to the manufacturer's protocol.

### Quantitative polymerase chain reaction

The qPCR was conducted similar to that described previously [54–56]. Briefly, total RNA was extracted using Trizol (Invitrogen, Carlsbad, CA, USA) from kidney cortex of rats and quantitative PCR was performed using SYBR Green Master Mix (Qiagen, Duesseldorf, Germany). The sequences of primers of TGF-β1,MCP-1 and P-selectin were as previously described [57, 58]. Each reaction was amplified in triplicate and relative quantification of mRNA levels was calculated based on the 2^−△△CT^method.

### Statistical analysis

All data from this study are expressed as the mean ± SEM. Results were analyzed by SPSS (Ver 18.0, Chicago, IL). The significant differences were analyzed by a one-way analysis of variances (ANOVA) analysis, followed by Turkey's multiple comparison tests. Categorical variables were presented as frequencies and Kruskal-Wallis test followed by the Mann-Whitney *U* test was employed for nonparametric data comparison. For all the statistical tests, *P* values < 0.05 was considered significantly different.

## References

[1] Balakumar P, Arora MK, Reddy J, Anand-Srivastava MB (2009). Pathophysiology of diabetic nephropathy: involvement of multifaceted signalling mechanism. J Cardiovasc Pharm Col.

[2] Gross JL, de Azevedo MJ, Silveiro SP, Canani LH, Caramori ML, Zelmanovitz T (2005). Diabetic nephropathy: diagnosis, prevention, and treatment. Diabetes care.

[3] Shaw JE, Sicree RA, Zimmet PZ (2010). Global estimates of the prevalence of diabetes for 2010 and 2030. Diabetes research and clinical practice.

[4] Wang F, Xing T, Wang N, Liu L (2012). Clinical significance of plasma CD146 and P-selectin in patients with type 2 diabetic nephropathy. Cytokine.

[5] Wang F, Huang B, Li J, Liu L, Wang N (2014). Renalase might be associated with hypertension and insulin resistance in Type 2 diabetes. Renal failure.

[6] Oztekin N, Baskan S, Evrim Kepekci S, Erim FB, Topcu G (2010). Isolation and analysis of bioactive diterpenoids in Salvia species (Salvia chionantha and Salvia kronenburgiii) by micellar electrokinetic capillary chromatography. Journal of pharmaceutical and biomedical analysis.

[7] Zhou L, Zuo Z, Chow MS (2005). Danshen: an overview of its chemistry, pharmacology, pharmacokinetics, and clinical use. Journal of clinical pharmacology.

[8] Wang AM, Sha SH, Lesniak W, Schacht J (2003). Tanshinone (Salviae miltiorrhizae extract) preparations attenuate aminoglycoside-induced free radical formation *in vitro* and ototoxicity *in vivo*. Antimicrobial agents and chemotherapy.

[9] Jang SI, Jeong SI, Kim KJ, Kim HJ, Yu HH, Park R, Kim HM, You YO (2003). Tanshinone IIA from Salvia miltiorrhiza inhibits inducible nitric oxide synthase expression and production of TNF-alpha, IL-1beta and IL-6 in activated RAW 264.7 cells. Planta medica.

[10] Wang X, Wei Y, Yuan S, Liu G, Lu Y, Zhang J, Wang W (2005). Potential anticancer activity of tanshinone IIA against human breast cancer. International journal of cancer.

[11] Fang J, Xu SW, Wang P, Tang FT, Zhou SG, Gao J, Chen JW, Huang HQ, Liu PQ (2010). Tanshinone II-A attenuates cardiac fibrosis and modulates collagen metabolism in rats with renovascular hypertension. Phytomedicine.

[12] Xia WJ, Yang M, Fok TF, Li K, Chan WY, Ng PC, Ng HK, Chik KW, Wang CC, Gu GJ, Woo KS, Fung KP (2005). Partial neuroprotective effect of pretreatment with tanshinone IIA on neonatal hypoxia-ischemia brain damage. Pediatric research.

[13] Kwak HB, Yang D, Ha H, Lee JH, Kim HN, Woo ER, Lee S, Kim HH, Lee ZH (2006). Tanshinone IIA inhibits osteoclast differentiation through down-regulation of c-Fos and NFATc1. Experimental & molecular medicine.

[14] Jiang C, Zhu W, Yan X, Shao Q, Xu B, Zhang M, Gong R (2016). Rescue therapy with Tanshinone IIA hinders transition of acute kidney injury to chronic kidney disease via targeting GSK3beta. Scientific reports.

[15] Jiang C, Shao Q, Jin B, Gong R, Zhang M, Xu B (2015). Tanshinone IIA Attenuates Renal Fibrosis after Acute Kidney Injury in a Mouse Model through Inhibition of Fibrocytes Recruitment. BioMed research international.

[16] Wu X, Liu L, Xie H, Liao J, Zhou X, Wan J, Yu K, Li J, Zhang Y (2012). Tanshinone IIA prevents uric acid nephropathy in rats through NF-kappaB inhibition. Planta medica.

[17] Liu X, Wang Y, Ma C, Zhang L, Wu W, Guan S, Yang M, Wang J, Jiang B, Guo DA (2011). Proteomic assessment of tanshinone IIA sodium sulfonate on doxorubicin induced nephropathy. The American journal of Chinese medicine.

[18] Ahn YM, Kim SK, Lee SH, Ahn SY, Kang SW, Chung JH, Kim SD, Lee BC (2010). Renoprotective effect of Tanshinone IIA, an active component of Salvia miltiorrhiza, on rats with chronic kidney disease. Phytotherapy research.

[19] Zhang X, He D, Xu L, Ling S (2012). Protective effect of tanshinone IIA on rat kidneys during hypothermic preservation. Molecular medicine reports.

[20] Kim SK, Jung KH, Lee BC (2009). Protective effect of Tanshinone IIA on the early stage of experimental diabetic nephropathy. Biological & pharmaceutical bulletin.

[21] Bakris GL (2008). Slowing nephropathy progression: focus on proteinuria reduction. Clinical journal of the American Society of Nephrology.

[22] Fioretto P, Mauer M (2007). Histopathology of diabetic nephropathy. Seminars in nephrology.

[23] Wada J, Makino H (2013). Inflammation and the pathogenesis of diabetic nephropathy. Clinical science.

[24] Lim AK, Tesch GH (2012). Inflammation in diabetic nephropathy. Mediators of inflammation.

[25] Shlipak M (2010). Diabetic nephropathy: preventing progression. BMJ clinical evidence.

[26] Brownlee M (2001). Biochemistry and molecular cell biology of diabetic complications. Nature.

[27] Hoffman BB, Sharma K, Ziyadeh FN (1998). Potential role of TGF-beta in diabetic nephropathy. Mineral and electrolyte metabolism.

[28] Paulini J, Higuti E, Bastos RM, Gomes SA, Rangel EB (2016). Mesenchymal Stem Cells as Therapeutic Candidates for Halting the Progression of Diabetic Nephropathy. Stem cells international.

[29] Tang J, Zhan C, Zhou J (2008). Effects of tanshinone IIA on transforming growth factor beta1-Smads signal pathway in renal interstitial fibroblasts of rats. J Huazhong Univ Sci Technology Med Sci.

[30] Giunti S, Tesch GH, Pinach S, Burt DJ, Cooper ME, Cavallo-Perin P, Camussi G, Gruden G (2008). Monocyte chemoattractant protein-1 has prosclerotic effects both in a mouse model of experimental diabetes and *in vitro* in human mesangial cells. Diabetologia.

[31] Park J, Ryu DR, Li JJ, Jung DS, Kwak SJ, Lee SH, Yoo TH, Han SH, Lee JE, Kim DK, Moon SJ, Kim K, Han DS (2008). MCP-1/CCR2 system is involved in high glucose-induced fibronectin and type IV collagen expression in cultured mesangial cells. Am J Physid Renal Physid.

[32] Chow FY, Nikolic-Paterson DJ, Ozols E, Atkins RC, Rollin BJ, Tesch GH (2006). Monocyte chemoattractant protein-1 promotes the development of diabetic renal injury in streptozotocin-treated mice. Kidney international.

[33] Chow F, Ozols E, Nikolic-Paterson DJ, Atkins RC, Tesch GH (2004). Macrophages in mouse type 2 diabetic nephropathy: correlation with diabetic state and progressive renal injury. Kidney international.

[34] Binder FP, Ernst B. E- (2011). P-selectin: differences, similarities and implications for the design of P-selectin antagonists. Chimia.

[35] Moulton V, Tsokos GC (2015). T cell signaling abnormalities in systemic autoimmunity explain aberrant immune cell function and provide rational targets for treatment. J Clin. Invest.

[36] Wang DT, Huang RH, Cheng X, Zhang ZH, Yang YJ, Lin X (2015). Tanshinone IIA attenuates renal fibrosis and inflammation via altering expression of TGF-beta/Smad and NF-kappaB signaling pathway in 5/6 nephrectomized rats. Int immunopharmaco.

[37] Miranda-Diaz AG, Pazarin-Villasenor L, Yanowsky-Escatell FG, Andrade-Sierra J (2016). Oxidative Stress in Diabetic Nephropathy with Early Chronic Kidney Disease. Journal of diabetes research.

[38] Brownlee M (2005). The pathobiology of diabetic complications: a unifying mechanism. Diabetes.

[39] Forbes JM, Cooper ME (2013). Mechanisms of diabetic complications. Physiological reviews.

[40] Lu Z, Wang F, Liang M (2017). SerpinC1/Antithrombin III in kidney related diseases. Clinical Science.

[41] Lu Z, Cheng D, Yin J, Wu R, Zhang G, Zhao Q, Wang N, Wang F, Liang M (2017). Antithrombin III Protects Against Contrast-Induced Nephropathy. Ebiomedicine.

[42] Lu Z, Yin J, Zhang G, Wu R, Zhao Q, Wang N, Yan C, Wang F (2017). Underestimated incidence of kidney disease in nonrenal outpatient. Ren Fail.

[43] Lu Z, Liu N, Wang F (2017). Epigenetic Regulations in Diabetic Nephropathy. Journal of diabetes research.

[44] Xue R, Gui D, Zheng L, Zhai R, Wang F, Wang N (2017). Mechanistic Insight and Management of Diabetic Nephropathy: Recent Progress and Future Perspective. Journal of diabetes research.

[45] Lu Z, Yin J, Bao H, Jiao Q, Wu H, Wu R, Xue Q, Wang N, Zhang Z, Wang F (2016). Coexistence of Acute Crescent Glomerulonephritis and IgG4-Related Kidney Disease. Case Rep Nephrol Dial.

[46] Wang F, Xing T, Li J, Zhang ZG, Wang N (2014). Coexisting glomerular IgA deposition and IgG-kappa multiple myeloma. Renal failure.

[47] Wang F, Xing T, Li J, Bai M, Hu R, Zhao Z, Tian S, Zhang Z, Wang N (2012). Renalase’s expression and distribution in renal tissue and cells. PloS one.

[48] Zhao Q, Yin J, Lu Z, Kong Y, Zhang G, Zhao B, Wang F (2016). Sulodexide Protects Contrast-Induced Nephropathy in Sprague-Dawley Rats. Cellular physiology and biochemistry.

[49] Yin J, Chen W, Ma F, Lu Z, Wu R, Zhang G, Wang N, Wang F Sulodexide pretreatment attenuates renal ischemia-reperfusion injury in rats. Oncotarget.

[50] Zhao B, Zhao Q, Li J, Xing T, Wang F, Wang N (2015). Renalase protects against contrast-induced nephropathy in Sprague-Dawley rats. PloS one.

[51] Wang F, Zhang G, Xing T, Lu Z, Li J, Peng C, Liu G, Wang N (2015). Renalase contributes to the renal protection of delayed ischaemic preconditioning via the regulation of hypoxia-inducible factor-1alpha. Journal of cellular and molecular medicine.

[52] Wang F, Zhang G, Lu Z, Geurts AM, Usa K, Jacob HJ, Cowley AW, Wang N, Liang M (2015). Antithrombin III/SerpinC1 insufficiency exacerbates renal ischemia/reperfusion injury. Kidney international.

[53] Wang F, Yin J, Lu Z, Zhang G, Li J, Xing T, Zhuang S, Wang N (2016). Limb ischemic preconditioning protects against contrast-induced nephropathy via renalase. EBioMedicine.

[54] Wang F, Li L, Xu H, Liu Y, Yang C, Cowley AW, Wang N, Liu P, Liang M (2014). Characteristics of long non-coding RNAs in the Brown Norway rat and alterations in the Dahl salt-sensitive rat. Scientific reports.

[55] Wang F, Zhang G, Zhou Y, Gui D, Li J, Xing T, Wang N (2014). Magnolin protects against contrast-induced nephropathy in rats via antioxidation and antiapoptosis. Oxidative medicine and cellular longevity.

[56] Wang F, Cai H, Zhao Q, Xing T, Li J, Wang N (2014). Epinephrine evokes renalase secretion via alpha-adrenoceptor/NF-kappaB pathways in renal proximal tubular epithelial cells. Kidney & blood pressure research.

[57] Zhou Y, Wang F, Hao L, Wang N (2013). Effects of magnoline on P-selectin’s expression in diabetic rats and its reno-protection. Kidney & blood pressure research.

[58] Yin J, Lu Z, Wang F, Jiang Z, Lu L, Miao N, Wang N (2016). Renalase attenuates hypertension, renal injury and cardiac remodelling in rats with subtotal nephrectomy. J Cell Mol Med.

